# Effect of treatment duration on the associations between three modern antidiabetic drugs and survival outcomes of lung cancer in China

**DOI:** 10.3389/fmolb.2025.1701515

**Published:** 2026-01-07

**Authors:** Zijia Chen, Xiaonan Wang, Zhongtao Zhang, Lu Yang, Chao Lei, Yupeng Di, Ye Huang, Yan Li

**Affiliations:** 1 Emergency Department, Xiyuan Hospital of Chinese Academy of Chinese Medical Sciences, Beijing, China; 2 Department of Respiratory Medicine, Hengshui Traditional Chinese Medicine Hospital, Hengshui, Hebei, China; 3 Second Clinical Medical College, Guangzhou University of Chinese Medicine, Guangzhou, China; 4 Guang’anmen Hospital of Chinese Academy of Chinese Medical Sciences, Beijing, China; 5 Air Force Medical Center, PLA, Beijing, China; 6 Clinical Lab, The Fourth Hospital of Hebei Medical University, Shijiazhuang, Hebei, China

**Keywords:** lung cancer, real world study, T2DM, survival, antidiabetic drugs

## Abstract

**Background:**

Some antidiabetic drugs have been shown to have tumor suppressor or activator properties. The associations between the treatment durations of three relatively new classes of antidiabetic medications, namely glucagon-like peptide-1 receptor agonists (GLP-1RA), dipeptidyl peptidase 4 inhibitors (DPP-4I), and sodium–glucose cotransporter 2 inhibitors (SGLT-2I), and lung cancer prognosis remain unclear.

**Methods:**

A retrospective analysis was conducted on 11,357 newly diagnosed lung cancer patients with type 2 diabetes; these patients were recruited from the National Healthcare Big Data (East) Center and were divided into three groups based on their use of DPP-4I, GLP-1RA, or SGLT-2I, along with categorization of their treatment durations. Cox proportional hazards models were employed to assess the associations between drug duration and survival outcomes, including progression-free survival (PFS) and overall survival (OS). The multivariable models were adjusted for covariates like age, sex, smoking status, biomarkers, and cancer treatments. Sensitivity analyses and Kaplan–Meier estimates were used to validate the findings.

**Results:**

In terms of the PFS, the highest quartile of GLP-1RA treatment (≥560 days) showed a lower incidence of cancer progression (hazard ratio (HR): 0.43; 95% confidence interval (CI): 0.18, 1.03), although the results were not statistically significant. DPP-4I and SGLT-2I showed less consistent trends. In terms of OS, GLP-1RA demonstrated a linear dose–response characteristic with reduced mortality risk over longer treatment durations, whereas DPP-4I and SGLT-2I showed non-linear associations. The sensitivity analyses confirmed these findings.

**Conclusion:**

Longer treatment durations of GLP-1RA, SGLT-2I, and DPP-4I reduced the risks of disease progression and mortality in lung cancer patients with type 2 diabetes. Among these drug classes, GLP-1RA showed consistent benefits while DPP-4I and SGLT-2I had non-linear associations, with shorter treatment durations being linked to higher risk.

## 1 Introduction

Lung cancer remains one of the most prevalent malignancies and a leading cause of cancer-related deaths globally (Torre et al., 2015), particularly among individuals with preexisting conditions like diabetes (Brabletz et al., 2018; Leiter et al., 2021; Yao et al., 2019). Diabetes, particularly type 2 diabetes (T2D), is a significant comorbidity that has been shown to influence the prognosis of various cancers, including lung cancer (Shahid et al., 2021). According to the Global Burden of Diseases, Injuries, and Risk Factors study, diabetes was the eighth leading cause of combined death and disability worldwide as of 2019, affecting nearly 460 million people across different demographics (GBD, 2019 Diseases and Injuries Collaborators, 2020).

Diabetes can impact the progression and outcomes of lung cancer through several mechanisms, including hyperinsulinemia, hyperglycemia, and chronic inflammation, all of which are linked to increased cell proliferation and cancer progression (García-Jiménez et al., 2014; Morss and Edelman, 2007). In particular, preexisting diabetes can adversely affect lung cancer outcomes by influencing clinical decisions regarding lung cancer treatment or response to treatment (van de Poll-Franse et al., 2007).

Although research has suggested that certain antidiabetic medications may positively affect both the risk and prognosis of lung cancer (Tian et al., 2016; Xu et al., 2015), extant studies have predominantly focused on the effects of metformin and thiazolidinediones (Lu et al., 2018; Fan et al., 2023). To date, there is limited research on how the treatment durations of relatively newer classes of antidiabetic medications, such as glucagon-like peptide-1 receptor agonists (GLP-1RA), dipeptidyl peptidase 4 inhibitors (DPP-4I), and sodium–glucose cotransporter 2 inhibitors (SGLT-2I), influence lung cancer prognosis. These drugs with their distinct action mechanisms are increasingly being used to manage T2D. However, their impacts on cancer progression, particularly in lung cancer patients, remain under investigation.

Given the increasing prevalence of diabetes, it is crucial to understand how the duration of antidiabetic medication usage can affect lung cancer survival outcomes. The present study aims to evaluate the effects of DPP-4I, SGLT-2I, and GLP-1RA treatment durations on lung cancer progression and mortality in diabetic patients to inform better prevention and therapeutic strategies.

## 2 Methods

### 2.1 Study population

A retrospective analysis was performed on electronic medical record data from 459,269 newly diagnosed lung cancer cases registered with the National Healthcare Big Data (East) Center between 1 January 2020 and 1 July 2023. The study was approved by the Ethics Committee of Hengshui Hospital of Chinese Medicine (2024-KY-18). The lung cancer diagnoses were identified using the codes from the 10th revision of the International Classification of Diseases (ICD-10: C34). Among these lung cancer cases, a total of 47,812 patients were also diagnosed with T2D based on the ICD-10 codes (ICD-10: E11 and E14.9).

From this group, we selected 12,648 individuals with documented use of any of the three classes of antidiabetic drugs GLP-1RA, DPP-4I, and SGLT-2I for further analysis. After excluding patients with survival or progression-free survival (PFS) of less than 60 days, the final study sample comprised 11,357 lung cancer patients with T2D. Of these, 5,027 participants had been using antidiabetic drugs for less than 28 days and were therefore categorized as patients without treatment. Based on drug usage patterns, the final samples were divided into three study groups for analyses as follows: DPP-4I group (N = 7,923 including 5,027 patients without treatment and 2,896 patients receiving DPP-4I treatment); GLP-1RA group (N = 5,529 including 5,027 patients without treatment and 502 patients receiving GLP-1RA treatment); SGLT-2I group (N = 9,283 including 5,027 patients without treatment and 4,256 patients receiving SGLT-2I treatment) (Supplementary Figure S1). In the sensitivity analysis, the participants who had been using antidiabetic drugs for less than 14 days were considered untreated, which resulted in a subset of 4,407 untreated participants; the number of treated individuals per group was then adjusted accordingly (Supplementary Figure S2).

### 2.2 Antidiabetic drug treatment duration

To define the usage durations of the antidiabetic drugs, patients who had received treatment based on any of the three drug classes considered herein (GLP-1RA, DPP-4I, and SGLT-2I) for at least 28 days were categorized as users of the corresponding drugs, in accordance with previous randomized controlled trials (Grunberger, 2014; Hemmingsen et al., 2017; Musso et al., 2012). In the primary analysis, patients who had been using each of the three types of drugs for less than 28 days were classified as non-users.

For each drug, the treatment duration was calculated as the difference between the dates of drug discontinuation and drug initiation. The patients were then grouped into quartiles based on their duration of use of each medication.

### 2.3 Lung cancer survival outcomes

The primary events of interest in this study were lung cancer progression and all-cause mortality. Accordingly, the survival outcomes were assessed using two widely recognized indices: PFS and overall survival (OS). Here, PFS is defined as the time from the initiation of treatment to the occurrence of either disease progression or death (Gyawali et al., 2022), and OS is defined as the time from the commencement of treatment to death from any cause (Lebwohl et al., 2009).

### 2.4 Covariate assessment

The smoking statuses of the participants were categorized into one of two groups, namely, smokers (including both current and former smokers) and non-smokers. The drinking statuses of the cohort were similarly classified as drinkers, encompassing both current and former drinkers, and non-drinkers. Venous fasting blood samples were analyzed for several biomarkers, including hemoglobin A1c (HbA1c), fibrinogen, prothrombin time, as well as three cancer biomarkers, namely, cytokeratin fragment 21-1 (CYFRA21-1), cancer antigen 125 (CA-125), and carcinoembryonic antigen (CEA). Additional data were collected on the family history of lung cancer, presence of other chronic diseases (such as kidney disease, hypertension, stroke, coronary artery disease, and pulmonary disease) as identified by their ICD-10 codes, metastasis to other organs (brain, bone, liver, and kidney), diabetic complications, and anticancer treatments (including chemotherapy, immunotherapy, radiotherapy, targeted therapy, and bevacizumab antiangiogenic therapy). The hypoglycemic therapies received by the patients were categorized into several types as insulin secretagogues, biguanides, glucuronide inhibitors, and thiazolidinediones. The pathological classifications for cancer were designated under five categories, namely, squamous cell carcinoma, adenocarcinoma, adenosquamous carcinoma, small cell carcinoma, and large cell carcinoma.

### 2.5 Statistical analysis

The baseline demographic and clinical characteristics as well as treatment durations of the three antidiabetic drugs were summarized using either frequencies and percentages or medians with interquartile ranges (IQRs) depending on the data distribution. Group comparisons were then performed using the Kruskal–Wallis H test for continuous variables and chi-squared test for categorical variables.

The associations between the duration of treatment with GLP-1RA, DPP-4I, or SGLT-2I and both disease progression and all-cause mortality were examined using Cox proportional hazards regression models. Accordingly, four models were employed for the analyses: Model 1 representing the crude model; Model 2 including adjustments for age and sex; Model 3 with further adjustments for smoking and drinking statuses; Model 4 including additional adjustments for HbA1c (binary <6.5% and ≥6.5%) (International Expert Committee, 2009), kidney disease, stroke, pulmonary disease, diabetic complications, prothrombin time (<10, 10–13, and >13 s) (Winter et al., 2017), CYFRA21-1 (binary ≤3.15 and >3.15 ng/mL) (Garcia-Valdecasas Gayo et al., 2020), CA-125 (binary ≤35 and >35 U/mL) (Ghosh et al., 2013), CEA (binary: <2.5 and ≥2.5 μg/L for non-smokers; <5 and ≥5 μg/L for smokers) (Ghosh et al., 2013), chemotherapy, immunotherapy, targeted therapy, biguanides, surgical treatment, type of pathology, and metastasis (binary yes/no).

The PFS and OS were evaluated using the Kaplan–Meier method, and the differences in the median survival times were assessed using the log-rank test. To investigate potential non-linearities in the relationships between treatment durations and outcomes, we applied three-knot restricted cubic splines by treating the treatment duration as a continuous variable.

For the sensitivity analyses, the users were redefined as those who had used the prescribed drugs for at least 14 continuous days, and the above analyses were repeated to ensure robustness of the results.

All statistical analyses were conducted using SAS software (version 9.4, SAS Institute Inc., Cary, NC, United States) and R software (version 4.4.1, https://www.r-project.org/). The statistical significance was defined as a two-sided *p*-value of <0.05.

## 3 Results

### 3.1 General characteristics of the study population

The baseline characteristics of lung cancer patients with T2D who received GLP-1RA (n = 5,529) are presented in Table 1, with stratification based on the duration of GLP-1RA treatment into quartiles. For additional details, the baseline characteristics of the patients who received DPP-4I and SGLT-2I therapies are provided in the Supplementary Material (Supplementary Tables S1, S2). Among these patients, 5,027 individuals did not receive any treatment. For patients receiving GLP-1RA, the median age was 68 years (IQR: 60, 74 years), where 39.6% were men, 23.58% were smokers, and 12.05% were drinkers. The median HbA1c level was 7.90% (IQR: 6.80%, 9.40%), and the patients had normal hemostatic functions in general. Compared with the untreated patients, the persons in the highest GLP-1RA treatment duration group were younger, had lower levels of cancer biomarkers, tended to use other hypoglycemic drugs, and mostly showed adenocarcinoma pathology (Table 1).

**TABLE 1:** Baseline characteristics of patients with different GLP-1RA treatment durations.

Variable	Total (n = 5529)	Without (n = 5027)	Quartile 1 (n = 124)	Quartile 2 (n = 127)	Quartile 3 (n = 125)	Quartile 4 (n = 126)	*p*-value
Age, years	68 (60, 74)	68 (61, 74)	58 (43, 67)	59 (48, 67)	62 (54, 70)	62.5 (54, 68.75)	<0.001
Male	2,191 (39.63)	1,983 (39.45)	56 (45.16)	44 (34.65)	55 (44.00)	53 (42.06)	0.369
Smokers	1,304 (23.58)	1,203 (23.93)	20 (16.13)	28 (22.05)	26 (20.80)	27 (21.43)	0.269
Drinkers	666 (12.05)	604 (12.02)	9 (7.26)	22 (17.32)	12 (9.60)	19 (15.08)	0.098
Family history	71 (1.28)	65 (1.29)	0 (0.00)	3 (2.36)	0 (0.00)	3 (2.38)	0.202
Kidney disease	337 (6.10)	293 (5.83)	11 (8.87)	12 (9.45)	14 (11.20)	7 (5.56)	0.032
Hypertension	3,355 (60.68)	3,036 (60.39)	66 (53.23)	77 (60.63)	88 (70.40)	88 (69.84)	0.014
Coronary heart disease	1,121 (20.27)	1,003 (19.95)	22 (17.74)	33 (25.98)	32 (25.60)	31 (24.60)	0.134
Stroke	1,522 (27.53)	1,384 (27.53)	31 (25.00)	25 (19.69)	36 (28.80)	46 (36.51)	0.050
Pulmonary disease	346 (6.26)	330 (6.56)	1 (0.81)	3 (2.36)	6 (4.80)	6 (4.76)	0.023
Diabetic complications	604 (10.92)	464 (9.23)	29 (23.39)	33 (25.98)	35 (28.00)	43 (34.13)	<0.001
HbA1c, %	7.90 (6.80, 9.40)	7.90 (6.80, 9.34)	8.44 (7, 10.12)	8.40 (7.21, 10.08)	8.40 (7.40, 9.90)	7.90 (6.70, 9.23)	<0.001
Fibrinogen, g/L	3.40 (2.71, 4.40)	3.42 (2.73, 4.44)	3.38 (2.75, 4.10)	3.44 (2.83, 4.38)	3.16 (2.57, 4.06)	3.09 (2.53, 3.82)	0.004
Prothrombin time, s	11.70 (10.90, 12.90)	11.70 (10.90, 12.90)	11.80 (10.90, 13.18)	11.55 (10.60, 13.22)	11.20 (10.60, 12.28)	11.50 (10.72, 12.56)	0.009
CYFRA21-1, ng/mL	5.52 (2.65, 12.43)	5.51 (2.67, 12.50)	6.34 (2.92, 12.14)	5.74 (2.08, 11.36)	5.44 (2.41, 12.76)	5.24 (2.29, 11.47)	0.431
CA-125, U/mL	93.53 (20.13, 191.28)	92.71 (19.81, 191.44)	75.38 (14.61, 189.40)	119.88 (41.99, 203.26)	105.51 (34.90, 191.18)	78.43 (15.50, 187.92)	0.139
CEA, μg/L	4.08 (2.20, 32.10)	4.31 (2.25, 38.52)	2.56 (1.87, 6.41)	3.24 (1.79, 8.86)	2.80 (1.76, 4.86)	2.65 (1.82, 3.53)	<0.001
Chemotherapy	1,790 (32.37)	1,697 (33.76)	17 (13.71)	33 (25.98)	21 (16.80)	22 (17.46)	<0.001
Radiotherapy	576 (10.42)	549 (10.92)	8 (6.45)	13 (10.24)	2 (1.60)	4 (3.17)	<0.001
Immunotherapy	422 (7.63)	404 (8.04)	4 (3.23)	8 (6.30)	2 (1.60)	4 (3.17)	0.005
Targeted therapy	549 (9.93)	533 (10.60)	4 (3.23)	4 (3.15)	4 (3.20)	4 (3.17)	<0.001
Bevacizumab antiangiogenic therapy	234 (4.23)	228 (4.54)	2 (1.61)	0 (0.00)	2 (1.60)	2 (1.59)	0.011
Insulin secretagogues	1,736 (31.40)	1,591 (31.65)	29 (23.39)	44 (34.65)	32 (25.60)	40 (31.75)	0.170
Biguanides	3,417 (61.80)	3,026 (60.19)	85 (68.55)	96 (75.59)	105 (84.00)	105 (83.33)	<0.001
Glucuronide inhibitors	2,078 (37.58)	1,868 (37.16)	37 (29.84)	54 (42.52)	51 (40.80)	68 (53.97)	<0.001
Thiazolidinedione	214 (3.87)	182 (3.62)	5 (4.03)	6 (4.72)	11 (8.80)	10 (7.94)	0.008
Surgical treatment	1,504 (27.20)	1,382 (27.49)	27 (21.77)	24 (18.90)	30 (24.00)	41 (32.54)	0.063
Type of pathology							0.013
Squamous cell carcinomas	1,217 (22.01)	1,132 (22.52)	17 (13.71)	24 (18.90)	24 (19.20)	20 (15.87)	
Adenocarcinoma	3,795 (68.64)	3,400 (67.63)	101 (81.45)	97 (76.38)	98 (78.40)	99 (78.57)	
Adenosquamous carcinoma	43 (0.78)	42 (0.84)	0 (0.00)	0 (0.00)	0 (0.00)	1 (0.79)	
Small cell carcinoma	458 (8.28)	437 (8.69)	6 (4.84)	6 (4.72)	3 (2.40)	6 (4.76)	
Large cell carcinoma	16 (0.29)	16 (0.32)	0 (0.00)	0 (0.00)	0 (0.00)	0 (0.00)	
Brain metastasis	78 (1.41)	74 (1.47)	1 (0.81)	1 (0.79)	2 (1.60)	0 (0.00)	0.780
Bone metastasis	184 (3.33)	181 (3.60)	0 (0.00)	2 (1.57)	1 (0.80)	0 (0.00)	0.003
Liver metastasis	73 (1.32)	69 (1.37)	0 (0.00)	1 (0.79)	3 (2.40)	0 (0.00)	0.362
Renal metastasis	47 (0.85)	46 (0.92)	0 (0.00)	0 (0.00)	1 (0.80)	0 (0.00)	0.792
Progression	994 (17.98)	962 (19.14)	6 (4.84)	15 (11.81)	6 (4.80)	5 (3.97)	<0.001
Progression-free survival, days	493 (260, 772)	480 (256, 770)	593 (270.5, 802.7)	434 (250, 740)	555 (377, 685)	705.5 (380.7, 806.5)	<0.001
Death	820 (14.83)	795 (15.81)	4 (3.23)	12 (9.45)	5 (4.00)	4 (3.17)	<0.001
Survival time, days	488 (252, 761)	484 (250.50, 762)	563 (247.25, 772.75)	416 (226.5, 716)	525 (347, 655)	675.5 (356.3, 776.5)	0.014

Abbreviations: CYFRA21-1, cytokeratin fragment 21-1; CA-125, cancer antigen 125; CEA, carcinoembryonic antigen.

### 3.2 Association between antidiabetic treatment duration and disease progression

The median PFS for GLP-1RA was 1.35 years, which generated 7,483 person-years of follow-up data, among which 994 patients had lung cancer progression. The median PFS values for DPP-4I and SGLT-2I were 1.35 and 1.33 years, respectively.

For GLP-1RA, the relationship between treatment duration and lung cancer progression exhibited a clear linear trend. Patients in the highest quartile of the GLP-1RA treatment duration (≥560 days) had the lowest incidence of cancer progression (incidence density: 1.93) and best risk reduction across all models. In Model 4, the hazard ratio (HR) for this quartile was 0.44 (95% confidence interval (CI): 0.16, 1.18), which reflected consistent benefits associated with longer treatment durations despite the lack of statistical significance in the fully adjusted analysis (Table 2).

**TABLE 2:** Associations between GLP-1RA, DPP-4I, and SGLT-2I treatment durations and disease progression.

Therapy	Case/Total	Incidence density^a^	Model 1	Model 2	Model 3	Model 4
GLP-1RA						
Without	962/5,027	13.23	1.00	1.00	1.00	1.00
Quartile 1 (<106 days)	6/124	3.16	0.23 (0.11, 0.53)	0.35 (0.16, 0.78)	0.36 (0.16, 0.80)	0.51 (0.23, 1.13)
Quartile 2 (106–292 days)	15/127	8.43	0.63 (0.38, 1.06)	0.78 (0.47, 1.29)	0.77 (0.46, 1.29)	0.89 (0.53, 1.50)
Quartile 3 (293–559 days)	6/125	3.20	0.24 (0.11, 0.53)	0.31 (0.14, 0.69)	0.31 (0.14, 0.69)	0.50 (0.22, 1.12)
Quartile 4 (≥560 days)	5/126	2.31	0.18 (0.07, 0.42)	0.23 (0.10, 0.56)	0.23 (0.10, 0.56)	0.43 (0.18, 1.03)
DPP-4I						
Without	962/5,027	13.23	1.00	1.00	1.00	1.00
Quartile 1 (<111 days)	185/718	17.97	1.36 (1.17, 1.60)	1.30 (1.11, 1.52)	1.28 (1.10, 1.50)	1.08 (0.92, 1.27)
Quartile 2 (111–302 days)	190/725	18.75	1.42 (1.21, 1.65)	1.40 (1.20, 1.64)	1.40 (1.19, 1.63)	1.22 (1.04, 1.43)
Quartile 3 (303–622 days)	148/726	13.33	1.00 (0.84, 1.19)	1.02 (0.86, 1.22)	1.01 (0.85, 1.20)	0.93 (0.78, 1.11)
Quartile 4 (≥623 days)	109/727	8.88	0.67 (0.55, 0.82)	0.70 (0.57, 0.85)	0.70 (0.57, 0.85)	0.86 (0.70, 1.05)
SGLT-2I						
Without	962/5,027	13.23	1.00	1.00	1.00	1.00
Quartile 1 (<126 days)	201/1,053	14.19	1.08 (0.92, 1.25)	1.07 (0.92, 1.25)	1.08 (0.92, 1.25)	1.02 (0.87, 1.18)
Quartile 2 (126–321 days)	225/1,075	15.56	1.17 (1.01, 1.35)	1.17 (1.01, 1.35)	1.17 (1.01, 1.35)	1.17 (1.01, 1.35)
Quartile 3 (322–607 days)	176/1,063	10.88	0.81 (0.69, 0.96)	0.83 (0.71, 0.98)	0.83 (0.71, 0.97)	0.90 (0.76, 1.06)
Quartile 4 (≥608 days)	126/1,065	6.94	0.53 (0.44, 0.63)	0.56 (0.46, 0.67)	0.56 (0.47, 0.68)	0.72 (0.59, 0.87)

^a^ The unit of incidence density is per 100 person-years.

Model 1 was the crude model.

Model 2 was adjusted for age and sex.

Model 3 was further adjusted for smoking and drinking statuses.

Model 4 was additionally adjusted for HbA1c (<6.5 and ≥6.5%), kidney disease, stroke, pulmonary disease, diabetic complications, prothrombin time (<10, 10–13, and >13 s), CYFRA21-1 (≤3.15 and >3.15 ng/mL), CA-125 (≤35 and >35 U/mL), CEA (for non-smokers: <2.5 and ≥2.5 μg/L; for smokers: <5 and ≥5 μg/L), chemotherapy, immunotherapy, targeted therapy, biguanides, surgical treatment, type of pathology, and metastasis (with and without).

In contrast, for DPP-4I and SGLT-2I, the associations between the corresponding treatment durations and lung cancer progression were not consistent with the dose–response relationships. For DPP-4I, the highest quartile of treatment duration (≥623 days) showed reduced risk of progression (incidence density: 5.90; HR: 0.65; 95% CI: 0.51, 0.83) in Model 4 but the lower quartiles did not exhibit any clear trends. Similarly, for SGLT-2I, the highest quartile of treatment duration (≥608 days) demonstrated the lowest risk of progression (incidence density: 4.85; HR: 0.64; 95% CI: 0.51, 0.80) in Model 4 but there was no consistent dose–response effect across the quartiles. The sensitivity analyses yielded similar results (Supplementary Table S3).

### 3.3 Association between antidiabetic treatment duration and death

The median OS for GLP-1RA was 1.35 years, which generated 7,468 person-years of follow-up data, with 820 patient deaths. The median OS values for DPP-4I and SGLT-2I were 1.39 and 1.37 years, respectively.

The GLP-1RA group exhibited a linear trend of decreasing mortality risk with longer treatment duration (HR continuously decreased as treatment duration increased), but this association did not reach statistical significance (*p* > 0.05); thus, the true benefits of this therapy require further verifications in large-sample prospective studies. The incidence density decreased from 11.04 cases per 100 person-years in untreated patients to 1.93 cases per 100 person-years in those receiving ≥560 days of treatment. Compared to the untreated patients, the HR of the longest treatment duration group was 0.44 (95% CI: 0.16, 1.18) in Model 4 (Table 3).

**TABLE 3:** Associations between GLP-1RA, DPP-4I, and SGLT-2I treatment durations and death.

Therapy	Case/Total	Incidence density^a^	Model 1	Model 2	Model 3	Model 4
GLP-1RA						
Without	795/5,027	11.04	1.00	1.00	1.00	1.00
Quartile 1 (<106 days)	4/124	2.21	0.20 (0.07, 0.54)	0.32 (0.12, 0.85)	0.33 (0.12, 0.87)	0.42 (0.16, 1.12)
Quartile 2 (106–292 days)	12/127	6.99	0.64 (0.36, 1.13)	0.81 (0.46, 1.44)	0.81 (0.46, 1.43)	0.86 (0.48, 1.54)
Quartile 3 (293–559 days)	5/125	2.80	0.26 (0.11, 0.62)	0.35 (0.15, 0.84)	0.35 (0.15, 0.85)	0.52 (0.21, 1.25)
Quartile 4 (≥560 days)	4/126	1.93	0.18 (0.07, 0.47)	0.25 (0.09, 0.66)	0.25 (0.09, 0.67)	0.44 (0.16, 1.18)
DPP-4I						
Without	795/5,027	11.04	1.00	1.00	1.00	1.00
Quartile 1 (<111 days)	140/718	13.24	1.20 (0.999, 1.43)	1.13 (0.94, 1.35)	1.11 (0.93, 1.33)	0.96 (0.80, 1.15)
Quartile 2 (111–302 days)	137/725	12.87	1.16 (0.97, 1.39)	1.15 (0.96, 1.38)	1.15 (0.96, 1.37)	1.00 (0.84, 1.20)
Quartile 3 (303–622 days)	108/726	9.56	0.86 (0.70, 1.05)	0.89 (0.73, 1.09)	0.88 (0.72, 1.08)	0.87 (0.71, 1.06)
Quartile 4 (≥623 days)	72/727	5.90	0.53 (0.41, 0.67)	0.55 (0.43, 0.70)	0.55 (0.43, 0.70)	0.65 (0.51, 0.83)
SGLT-2I						
Without	795/5,027	11.04	1.00	1.00	1.00	1.00
Quartile 1 (<126 days)	141/1,053	9.86	0.90 (0.75, 1.08)	0.89 (0.75, 1.07)	0.89 (0.75, 1.07)	0.83 (0.69, 0.99)
Quartile 2 (126–321 days)	164/1,075	11.21	1.02 (0.86, 1.20)	1.03 (0.87, 1.22)	1.03 (0.87, 1.22)	1.03 (0.87, 1.22)
Quartile 3 (322–607 days)	132/1,063	8.21	0.74 (0.62, 0.89)	0.77 (0.64, 0.93)	0.77 (0.64, 0.93)	0.82 (0.68, 0.99)
Quartile 4 (≥608 days)	87/1,065	4.85	0.43 (0.35, 0.54)	0.49 (0.39, 0.61)	0.49 (0.39, 0.61)	0.64 (0.51, 0.80)

^a^ The unit of incidence density is per 100 person-years.

Model 1 was the crude model.

Model 2 was adjusted for age and sex.

Model 3 was further adjusted for smoking and drinking statuses.

Model 4 was additionally adjusted for HbA1c (<6.5 and ≥6.5%), kidney disease, stroke, pulmonary disease, diabetic complications, prothrombin time (<10, 10–13, and >13 s), CYFRA21-1 (≤3.15 and >3.15 ng/mL), CA-125 (≤35 and >35 U/mL), CEA (for non-smokers: <2.5 and ≥2.5 μg/L; for smokers: <5 and ≥5 μg/L), chemotherapy, immunotherapy, targeted therapy, biguanides, surgical treatment, type of pathology, and metastasis (with and without).

For the DPP-4I group, the incidence density decreased from 13.24 to 5.90 cases per 100 person-years with longer treatment. However, this association was not dose-dependent, with HRs ranging from 1.16 (95% CI: 0.97, 1.39) in the second quartile to 0.53 (95% CI: 0.41, 0.67) in the fourth quartile. Similarly, the SGLT-2I group showed reduced incidence density from 11.04 to 4.85 cases per 100 person-years; the HR was 0.43 (95% CI: 0.35, 0.54) for the highest quartile, but the dose–response effect was less consistent along with weaker associations in the lower quartiles. The sensitivity analyses yielded similar results (Supplementary Table S4).

### 3.4 Non-linear associations between treatment durations and survival outcomes

**FIGURE 1:**
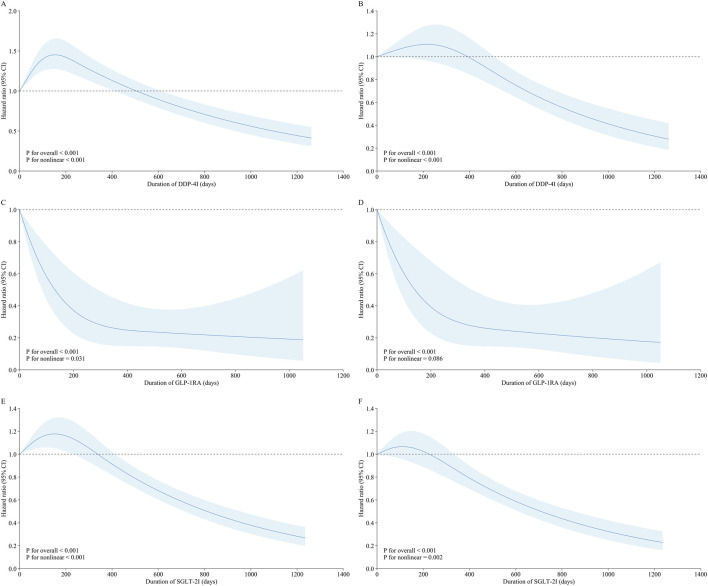
Dose–response relationships between **(A, B)** DPP-4I, **(C, D)** GLP-1RA, and **(E, F)** SGLT-2I drug classes and lung cancer progression as well as mortality. **(A, C, E)** Dose–response relationships between the treatment durations of the three classes of drugs and lung cancer progression. **(B, D, F)** Dose–response relationships between the treatment durations of the three classes of drugs and death (sensitivity analysis).

The restricted cubic spline analysis revealed non-linearities in the PFS for all three medications, with *p*-values <0.05. For the GLP-1RA group, the HR decreased with increasing treatment duration, indicating a linear benefit. In contrast, the DPP-4I and SGLT-2I groups showed HRs that initially increased and then decreased. In terms of OS, non-linearities were observed only for the DPP-4I and SGLT-2I groups (*p* < 0.05); both these classes of drugs demonstrated HRs that initially increased before declining. In contrast, the GLP-1RA group did not show significant non-linearity (*p* > 0.05), and the HR continuously decreased as treatment duration increased (Figure 1). The sensitivity analyses yielded similar results (Supplementary Figure S3).

## 4 Discussion

In this study, we observed that the highest quartiles of treatment duration in the GLP-1RA, SGLT-2I, and DPP-4I groups showed substantially reduced risks of disease progression and mortality for lung cancer patients with T2D. However, for the DPP-4I and SGLT-2I groups, the relationships between treatment durations and cancer outcomes were non-linear. Specifically, patients in the first and second quartiles of treatment duration showed increased risk of lung cancer progression. In contrast, the GLP-1RA group demonstrated consistent benefits, with longer treatment durations correlating with reduced progression and mortality risks.

Our findings align with the results of existing research, suggesting that diabetes medications can influence cancer outcomes. Research has shown that metformin, a common antidiabetic drug, may reduce cancer risk and improve survival outcomes (Xu et al., 2015; Xi et al., 2018). However, our study extends this knowledge by evaluating newer classes of antidiabetic drugs and their treatment durations. Specifically, recent findings indicate that GLP-1RA drugs inhibit lung cancer cell proliferation both *in vitro* and *in vivo*, underscoring their potential significance for managing diabetes in lung cancer patients (Pu et al., 2023). Similarly, SGLT-2I drugs have recently been shown to have anticancer effects, and their expression has been confirmed in many cancer cell lines, including lung cancer (Dutka et al., 2022; Villani et al., 2016). However, the impacts of DPP-4I drugs on lung cancer remain debatable (Zou et al., 2020); some retrospective studies have suggested that DPP-4I treatment may either have neutral effects or offer protection against cancer outcomes (Bishnoi et al., 2019; Shah et al., 2020; Ali et al., 2019), whereas a nationwide study in Hungary showed that DPP-4I users have higher mortality from cancer compared with the SGLT-2I cohort (Sütő et al., 2021). Our findings offer new insights to these earlier reports: short-term use of DPP-4I drugs (less than 1 year) may increase the risks of lung cancer progression and death, whereas long-term use may reduce these risks. These results highlight the need for further epidemiological and clinical trials to better understand the roles of DPP-4I drugs and their treatment durations in the prognosis of lung cancer patients with diabetes.

To the best of our knowledge, this is the first study that reports the effects of treatment durations of GLP-1RA, SGLT-2I, and DPP-4I classes of drugs on the survival outcomes of lung cancer. The observed benefits associated with prolonged GLP-1RA treatment, along with the non-linear effects of DPP-4I and SGLT-2I, highlight the complexity of these drug interactions with cancer biology. These findings may be attributed to the distinct mechanisms of these medications. Liraglutide (GLP-1RA) has been shown to inhibit lung cancer cell proliferation, migration, and epithelial–mesenchymal transitions (EMTs) *in vitro* and *in vivo*. EMT is a key process in cancer metastasis and is significantly suppressed by liraglutide, which also alleviates lung damage and reduces endoplasmic reticulum stress (Pu et al., 2023). This mechanistic understanding helps explain the consistent protective effects of GLP-1RA drugs observed in our study.

In addition to glucose transporters, sodium–glucose cotransporter 2 (SGLT2) was found to be expressed in human pancreatic, prostate, and lung tumors (Scafoglio et al., 2015; Scafoglio et al., 2018). A study showed that canagliflozin, an SGLT2 inhibitor, reduces tumor growth and enhances survival in a lung adenocarcinoma mouse model by inhibiting SGLT2 (Scafoglio et al., 2018). This suggests that SGLT2 could be a potential target in early-stage lung adenocarcinoma. However, our study could not assess the effects of SGLT-2I drugs in early versus advanced cancer stages. The lack of observable benefits from short-term SGLT-2I use may be attributed to the advanced stage of cancer in our cohort. Hence, further research is needed to explore the efficacy of SGLT-2I in the early stages of lung cancer.

The present study benefits from utilizing a large-scale electronic medical record database, which helps minimize selection bias and provides extensive pharmacological data for exploring the link between antidiabetic drugs and lung cancer prognosis. However, several limitations must be acknowledged. The retrospective nature of the study limits our ability to draw causal inferences about the impacts of treatment durations of different antidiabetic drugs on survival outcomes. Furthermore, patients with longer treatment durations may inherently have less severe disease, as those with more advanced disease may not tolerate long-term treatment. Although we adjusted for baseline severity, this inherent bias could still influence the results. Sensitivity analyses were performed to explore the impact of treatment duration further. Additionally, while the overall sample size exceeds 5,000 for each class of medication, the number of patients on specific drugs, such as GLP-1RA, is relatively small (fewer than 500), which could potentially affect the accuracy of the results. This may explain why the point estimates remained stable but lacked statistical significance despite the adjustment for numerous confounders. Nevertheless, our findings were consistent in the sensitivity analyses.

Although the patients in the highest quartile of GLP-1RA treatment duration showed a nominally lower HR, the 95% CI crossed unity, indicating non-significance. Thus, our findings should be interpreted as exploratory signals rather than definitive evidence and must be further validated in larger or prospective cohorts. Another important limitation of this study is the absence of complete tumor staging information in the current database. Although the National Healthcare Big Data (East) Center includes demographic, diagnostic, medication, and survival data, the tumor, node, and metastasis (TNM) stage was not consistently recorded across all participating institutions, particularly the primary hospitals. In future studies, we plan to integrate pathology-linked registries or hospital-based cancer databases to obtain more comprehensive staging information and thereby enhance the robustness of our analyses.

In conclusion, the present study demonstrates that long-term use (more than 1 year) of GLP-1RA, SGLT-2I, and DPP-4I classes of drugs may have great potential in lung cancer prognosis, while the short-term treatment durations of SGLT-2I and DPP-4I monotherapy may afford pro-cancer effects.

## Data Availability

The raw data supporting the conclusions of this article will be made available by the authors, without undue reservation.
