# Supplementary material for: Effect of treatment duration on the associations between three modern antidiabetic drugs and survival outcomes of lung cancer in China
Source: Front Mol Biosci. 2026 Jan 7;12:1701515. doi: 10.3389/fmolb.2025.1701515 (PMC12819283; doi:10.3389/fmolb.2025.1701515)
Supplement: Supplementary file 1 [file Supplementaryfile1.docx]

**Supplementary Materials**

**Effect of treatment duration on the association between three modern anti-diabetic drugs and survival outcomes of lung cancer in China**

Table S1 Baseline characteristics of patients with different DDP-4 treatment durations.

| Variables | Total (n = 7923) | Without  (n = 5027) | Quartile 1  (n = 718) | Quartile 2  (n = 725) | Quartile 3  (n = 726) | Quartile 4  (n = 727) | *P-value* |
| --- | --- | --- | --- | --- | --- | --- | --- |
| Age, years | 68 (60, 74) | 68 (61, 74) | 67.50 (60, 73) | 67 (60, 73) | 67 (60, 73) | 68 (60.5, 74) | **0.036** |
| Males | 4864 (61.39) | 3044 (60.55) | 497 (69.22) | 462 (63.72) | 449 (61.85) | 412 (56.67) | **<0.001** |
| Smoked | 1982 (25.02) | 1203 (23.93) | 219 (30.50) | 191 (26.34) | 198 (27.27) | 171 (23.52) | **0.001** |
| Drank | 986 (12.44) | 604 (12.02) | 94 (13.09) | 92 (12.69) | 106 (14.60) | 90 (12.38) | 0.371 |
| Family history | 109 (1.38) | 65 (1.29) | 14 (1.95) | 9 (1.24) | 11 (1.52) | 10 (1.38) | 0.699 |
| Kidney disease | 530 (6.69) | 293 (5.83) | 52 (7.24) | 61 (8.41) | 61 (8.40) | 63 (8.67) | **0.001** |
| Hypertension | 4854 (61.26) | 3036 (60.39) | 422 (58.77) | 455 (62.76) | 465 (64.05) | 476 (65.47) | **0.018** |
| Coronary heart disease | 1626 (20.52) | 1003 (19.95) | 119 (16.57) | 151 (20.83) | 160 (22.04) | 193 (26.55) | **<0.001** |
| Stroke | 2185 (27.58) | 1384 (27.53) | 192 (26.74) | 205 (28.28) | 212 (29.20) | 192 (26.41) | 0.756 |
| Pulmonary disease | 564 (7.12) | 330 (6.56) | 55 (7.66) | 66 (9.10) | 68 (9.37) | 45 (6.19) | **0.009** |
| Diabetic complications | 868 (10.96) | 464 (9.23) | 82 (11.42) | 91 (12.55) | 108 (14.88) | 123 (16.92) | **<0.001** |
| HbA1c, % | 7.80 (6.71, 9.21) | 7.90 (6.80, 9.34) | 7.92 (6.80, 9.31) | 7.60 (6.52, 9.10) | 7.66 (6.61, 9.00) | 7.47 (6.60, 8.80) | **<0.001** |
| Fibrinogen, g/L | 3.39 (2.70, 4.40) | 3.42 (2.73, 4.44) | 3.41 (2.73, 4.51) | 3.46 (2.72, 4.48) | 3.29 (2.62, 4.20) | 3.21 (2.59, 4.11) | **<0.001** |
| Prothrombin time, s | 11.63 (10.90, 12.90) | 11.70 (10.90, 12.90) | 11.70 (10.90, 13.00) | 11.70 (10.94, 12.88) | 11.60 (10.80, 12.70) | 11.50 (10.81, 12.70) | **0.038** |
| CYFRA21-1, ng/mL | 5.20 (2.62, 12.17) | 5.51 (2.67, 12.50) | 5.00 (2.58, 11.83) | 5.33 (2.57, 12.04) | 4.50 (2.51, 11.22) | 4.40 (2.42, 10.38) | **<0.001** |
| CA-125, U/mL | 91.63 (19.60, 190.56) | 92.71 (19.81, 191.44) | 90.37 (19.26, 179.19) | 92.25 (21.30, 189.86) | 84.06 (19.00, 197.52) | 84.73 (18.51, 182.41) | 0.775 |
| CEA, μg/L | 4.01 (2.15, 26.93) | 4.31 (2.25, 38.52) | 4.13 (2.20, 20.74) | 3.97 (2.12, 19.60) | 3.41 (1.97, 16.12) | 3.04 (1.85, 7.54) | **<0.001** |
| Chemotherapy | 2941 (37.12) | 1697 (33.76) | 351 (48.89) | 326 (44.97) | 308 (42.42) | 259 (35.63) | **<0.001** |
| Radiotherapy | 935 (11.80) | 549 (10.92) | 110 (15.32) | 107 (14.76) | 91 (12.53) | 78 (10.73) | **<0.001** |
| Immunotherapy | 714 (9.01) | 404 (8.04) | 90 (12.53) | 91 (12.55) | 83 (11.43) | 46 (6.33) | **<0.001** |
| Targeted therapy | 920 (11.61) | 533 (10.60) | 109 (15.18) | 106 (14.62) | 102 (14.05) | 70 (9.63) | **<0.001** |
| Bevacizumab anti-angiogenic therapy | 394 (4.97) | 228 (4.54) | 54 (7.52) | 41 (5.66) | 46 (6.34) | 25 (3.44) | **<0.001** |
| Insulin secretagogues | 2649 (33.43) | 1591 (31.65) | 256 (35.65) | 248 (34.21) | 269 (37.05) | 285 (39.20) | **<0.001** |
| Biguanides | 4896 (61.79) | 3026 (60.19) | 449 (62.53) | 445 (61.38) | 468 (64.46) | 508 (69.88) | **<0.001** |
| Glucuronide inhibitors | 3148 (39.73) | 1868 (37.16) | 321 (44.71) | 295 (40.69) | 317 (43.66) | 347 (47.73) | **<0.001** |
| Thiazolidinedione | 348 (4.39) | 182 (3.62) | 38 (5.29) | 27 (3.72) | 37 (5.10) | 64 (8.80) | **<0.001** |
| Surgical treatment | 2318 (29.26) | 1382 (27.49) | 205 (28.55) | 212 (29.24) | 238 (32.78) | 281 (38.65) | **<0.001** |
| Type of pathology |  |  |  |  |  |  | **0.003** |
| Squamous cell carcinomas | 1796 (22.67) | 1132 (22.52) | 196 (27.30) | 182 (25.10) | 151 (20.80) | 135 (18.57) |  |
| Adenocarcinoma | 5329 (67.26) | 3400 (67.63) | 439 (61.14) | 460 (63.45) | 503 (69.28) | 527 (72.49) |  |
| Adenosquamous carcinoma | 66 (0.83) | 42 (0.84) | 8 (1.11) | 8 (1.10) | 3 (0.41) | 5 (0.69) |  |
| Small cell carcinoma | 709 (8.95) | 437 (8.69) | 75 (10.45) | 71 (9.79) | 68 (9.37) | 58 (7.98) |  |
| Large cell carcinoma | 23 (0.29) | 16 (0.32) | 0 (0.00) | 4 (0.55) | 1 (0.14) | 2 (0.28) |  |
| Brain metastasis | 106 (1.34) | 74 (1.47) | 13 (1.81) | 7 (0.97) | 8 (1.10) | 4 (0.55) | 0.172 |
| Bone metastasis | 294 (3.71) | 181 (3.60) | 30 (4.18) | 35 (4.83) | 32 (4.41) | 16 (2.20) | 0.067 |
| Liver metastasis | 114 (1.44) | 69 (1.37) | 11 (1.53) | 15 (2.07) | 11 (1.52) | 8 (1.10) | 0.584 |
| Renal metastasis | 59 (0.74) | 46 (0.92) | 3 (0.42) | 4 (0.55) | 6 (0.83) | 0 (0.00) | 0.064 |
| Progression | 1594 (20.12) | 962 (19.14) | 185 (25.77) | 190 (26.21) | 148 (20.39) | 109 (14.99) | **<0.001** |
| Progression-free survival, days | 500 (262, 785) | 480 (256, 770) | 484 (232, 796) | 454 (244, 750) | 529.5 (323, 768.75) | 643 (321, 890.5) | **<0.001** |
| Death | 1252 (15.80) | 795 (15.81) | 140 (19.50) | 137 (18.90) | 108 (14.88) | 72 (9.90) | **<0.001** |
| Survival time, days | 507 (262, 785.5) | 484 (250.5, 762) | 510 (244.25, 820) | 497 (252, 804) | 545.5 (326, 782.75) | 655 (324, 876) | **<0.001** |

Abbreviations: CA-125: Carbohydrate antigen 125; CEA: Carcinoembryonic antigen.

Table S2. Baseline characteristics of patients with different SGLT-2 treatment durations.

| Variables | Total (n = 9283) | Without  (n = 5027) | Quartile 1  (n = 1053) | Quartile 2  (n = 1075) | Quartile 3  (n = 1063) | Quartile 4  (n = 1065) | *P-value* |
| --- | --- | --- | --- | --- | --- | --- | --- |
| Age, years | 68 (60, 74) | 68 (61, 74) | 67 (59, 73) | 68 (59, 74) | 67 (59, 73) | 66 (59, 73) | **<0.001** |
| Males | 3460 (37.27) | 1983 (39.45) | 352 (33.43) | 378 (35.16) | 359 (33.77) | 388 (36.43) | **<0.001** |
| Smoked | 2286 (24.63) | 1203 (23.93) | 274 (26.02) | 267 (24.84) | 281 (26.43) | 261 (24.51) | 0.364 |
| Drank | 1179 (12.70) | 604 (12.02) | 135 (12.82) | 125 (11.63) | 161 (15.15) | 154 (14.46) | **0.018** |
| Family history | 122 (1.31) | 65 (1.29) | 11 (1.04) | 9 (0.84) | 23 (2.16) | 14 (1.31) | 0.078 |
| Kidney disease | 565 (6.09) | 293 (5.83) | 71 (6.74) | 70 (6.51) | 59 (5.55) | 72 (6.76) | 0.541 |
| Hypertension | 5859 (63.12) | 3036 (60.39) | 663 (62.96) | 695 (64.65) | 725 (68.20) | 740 (69.48) | **<0.001** |
| Coronary heart disease | 2320 (24.99) | 1003 (19.95) | 277 (26.31) | 306 (28.47) | 360 (33.87) | 374 (35.12) | **<0.001** |
| Stroke | 2757 (29.70) | 1384 (27.53) | 325 (30.86) | 330 (30.70) | 355 (33.40) | 363 (34.08) | **<0.001** |
| Pulmonary disease | 621 (6.69) | 330 (6.56) | 79 (7.50) | 70 (6.51) | 77 (7.24) | 65 (6.10) | 0.662 |
| Diabetic complications | 1075 (11.58) | 464 (9.23) | 94 (8.93) | 125 (11.63) | 174 (16.37) | 218 (20.47) | **<0.001** |
| HbA1c, % | 7.90 (6.80, 9.30) | 7.90 (6.80, 9.34) | 7.97 (6.70, 9.40) | 7.99 (6.81, 9.36) | 7.98 (6.90, 9.30) | 7.70 (6.76, 9.14) | 0.134 |
| Fibrinogen, g/L | 3.36 (2.70, 4.37) | 3.42 (2.73, 4.44) | 3.27 (2.64, 4.32) | 3.36 (2.70, 4.40) | 3.32 (2.68, 4.25) | 3.20 (2.60, 4.12) | **<0.001** |
| Prothrombin time, s | 11.60 (10.90, 12.80) | 11.70 (10.90, 12.90) | 11.60 (10.90, 12.80) | 11.60 (11, 12.67) | 11.40 (10.80, 12.68) | 11.60 (10.90, 12.80) | **0.012** |
| CYFRA21-1, ng/mL | 5.14 (2.57, 11.94) | 5.51 (2.67, 12.50) | 5.23 (2.56, 12.20) | 4.59 (2.38, 10.91) | 4.77 (2.57, 10.99) | 4.45 (2.42, 10.28) | **<0.001** |
| CA-125, U/mL | 89.96 (18.48, 187.91) | 92.71 (19.81, 191.44) | 92.50 (16.70, 184.77) | 87.84 (19.10, 187.61) | 83.62 (16.89, 181.09) | 79.70 (16.20, 180.66) | **0.044** |
| CEA, μg/L | 3.87 (2.12, 23.88) | 4.31 (2.25, 38.52) | 3.90 (2.16, 24.48) | 3.68 (2.16, 16.76) | 3.33 (1.95, 9.91) | 2.97 (1.86, 7.28) | **<0.001** |
| Chemotherapy | 3309 (35.65) | 1697 (33.76) | 438 (41.60) | 426 (39.63) | 393 (36.97) | 355 (33.33) | **<0.001** |
| Radiotherapy | 1030 (11.10) | 549 (10.92) | 131 (12.44) | 133 (12.37) | 121 (11.38) | 96 (9.01) | 0.071 |
| Immunotherapy | 753 (8.11) | 404 (8.04) | 93 (8.83) | 95 (8.84) | 91 (8.56) | 70 (6.57) | 0.267 |
| Targeted therapy | 964 (10.38) | 533 (10.60) | 117 (11.11) | 115 (10.70) | 109 (10.25) | 90 (8.45) | 0.261 |
| Bevacizumab anti-angiogenic therapy | 409 (4.41) | 228 (4.54) | 53 (5.03) | 46 (4.28) | 49 (4.61) | 33 (3.10) | 0.227 |
| Insulin secretagogues | 3226 (34.75) | 1591 (31.65) | 402 (38.18) | 404 (37.58) | 399 (37.54) | 430 (40.38) | **<0.001** |
| Biguanides | 6084 (65.54) | 3026 (60.19) | 692 (65.72) | 773 (71.91) | 778 (73.19) | 815 (76.53) | **<0.001** |
| Glucuronide inhibitors | 3744 (40.33) | 1868 (37.16) | 413 (39.22) | 455 (42.33) | 483 (45.44) | 525 (49.30) | **<0.001** |
| Thiazolidinedione | 442 (4.76) | 182 (3.62) | 54 (5.13) | 64 (5.95) | 58 (5.46) | 84 (7.89) | **<0.001** |
| Surgical treatment | 2662 (28.68) | 1382 (27.49) | 280 (26.59) | 312 (29.02) | 317 (29.82) | 371 (34.84) | **<0.001** |
| Type of pathology |  |  |  |  |  |  | **0.046** |
| Squamous cell carcinomas | 2093 (22.55) | 1132 (22.52) | 264 (25.07) | 251 (23.35) | 224 (21.07) | 222 (20.85) |  |
| Adenocarcinoma | 6303 (67.90) | 3400 (67.63) | 685 (65.05) | 709 (65.95) | 743 (69.90) | 766 (71.92) |  |
| Adenosquamous carcinoma | 73 (0.79) | 42 (0.84) | 6 (0.57) | 8 (0.74) | 13 (1.22) | 4 (0.38) |  |
| Small cell carcinoma | 785 (8.46) | 437 (8.69) | 93 (8.83) | 105 (9.77) | 79 (7.43) | 71 (6.67) |  |
| Large cell carcinoma | 29 (0.31) | 16 (0.32) | 5 (0.47) | 2 (0.19) | 4 (0.38) | 2 (0.19) |  |
| Brain metastasis | 117 (1.26) | 74 (1.47) | 19 (1.80) | 7 (0.65) | 8 (0.75) | 9 (0.85) | **0.024** |
| Bone metastasis | 329 (3.54) | 181 (3.60) | 47 (4.46) | 38 (3.53) | 33 (3.10) | 30 (2.82) | 0.298 |
| Liver metastasis | 125 (1.35) | 69 (1.37) | 11 (1.04) | 17 (1.58) | 21 (1.98) | 7 (0.66) | 0.086 |
| Renal metastasis | 63 (0.68) | 46 (0.92) | 5 (0.47) | 6 (0.56) | 5 (0.47) | 1 (0.09) | **0.025** |
| Progression | 1690 (18.21) | 962 (19.14) | 201 (19.09) | 225 (20.93) | 176 (16.56) | 126 (11.83) | **<0.001** |
| Progression-free survival, days | 494 (266.50, 775) | 480 (256, 770) | 427 (219, 732) | 411 (254, 715) | 521 (337, 758) | 669 (344, 864) | **<0.001** |
| Death | 1319 (14.21) | 795 (15.81) | 141 (13.39) | 164 (15.26) | 132 (12.42) | 87 (8.17) | **<0.001** |
| Survival time, days | 499 (262, 770) | 484 (250.50, 762) | 438 (215, 748) | 434 (245.50, 733.50) | 523 (330, 750) | 670 (341, 848) | **<0.001** |

Abbreviations: CA-125: Carbohydrate antigen 125; CEA: Carcinoembryonic antigen.

Table S3. The association between GLP-1RA, DDP-4I, and SGLT-2I treatment duration and progression (Sensitivity analysis).

| Therapy | Case/Total | Incidence density* | Model 1 | Model 2 | Model 3 | Model 4 |
| --- | --- | --- | --- | --- | --- | --- |
| **GLP-1RA** |  |  |  |  |  |  |
| Without | 834/4407 | 13.19 | 1.00 | 1.00 | 1.00 | 1.00 |
| Quartile 1 (<106 days) | 7/136 | 3.22 | **0.25 (0.12, 0.52)** | **0.36 (0.17, 0.76)** | **0.37 (0.17, 0.77)** | 0.51 (0.24, 1.08) |
| Quartile 2 (106-292 days) | 15/134 | 8.13 | 0.62 (0.37, 1.03) | 0.79 (0.47, 1.32) | 0.79 (0.48, 1.33) | 0.97 (0.58, 1.64) |
| Quartile 3 (293-559 days) | 7/138 | 3.46 | **0.26 (0.12, 0.54)** | **0.32 (0.15, 0.68)** | **0.32 (0.15, 0.68)** | **0.50 (0.21, 0.95)** |
| Quartile 4 (≥560 days) | 6/136 | 2.54 | **0.19 (0.09, 0.43)** | **0.25 (0.11, 0.56)** | **0.26 (0.11, 0.57)** | 0.47 (0.21, 1.05) |
| **DDP-4I** |  |  |  |  |  |  |
| Without | 834/4407 | 13.19 | 1.00 | 1.00 | 1.00 | 1.00 |
| Quartile 1 (<111 days) | 199/807 | 16.28 | **1.24 (1.06, 1.45)** | **1.18 (1.01, 1.38)** | **1.18 (1.01, 1.38)** | 1.04 (0.89, 1.21) |
| Quartile 2 (111-302 days) | 208/809 | 18.74 | **1.43 (1.22, 1.66)** | **1.40 (1.20, 1.63)** | **1.39 (1.19, 1.62)** | **1.17 (1.004, 1.36)** |
| Quartile 3 (303-622 days) | 171/810 | 13.85 | 1.04 (0.88, 1.23) | 1.05 (0.89, 1.24) | 1.04 (0.88, 1.23) | 0.96 (0.81, 1.13) |
| Quartile 4 (≥623 days) | 130/811 | 9.58 | **0.73 (0.61, 0.88)** | **0.75 (0.63, 0.91)** | **0.75 (0.63, 0.90)** | 0.88 (0.73, 1.06) |
| **SGLT-2I** |  |  |  |  |  |  |
| Without | 834/4407 | 13.19 | 1.00 | 1.00 | 1.00 | 1.00 |
| Quartile 1 (<126 days) | 225/1159 | 13.99 | 1.06 (0.92, 1.23) | 1.05 (0.90, 1.21) | 1.04 (0.90, 1.21) | 1.04 (0.89, 1.20) |
| Quartile 2 (126-321 days) | 243/1161 | 15.57 | **1.18 (1.02, 1.36)** | **1.17 (1.02, 1.36)** | **1.19 (1.02, 1.36)** | 1.12 (0.97, 1.29) |
| Quartile 3 (322-607 days) | 193/1158 | 11.10 | **0.83 (0.71, 0.98)** | **0.84 (0.72, 0.99)** | **0.84 (0.72, 0.99)** | 0.90 (0.77, 1.05) |
| Quartile 4 (≥608 days) | 140/1162 | 7.13 | **0.54 (0.45, 0.65)** | **0.57 (0.48, 0.69)** | **0.57 (0.48, 0.69)** | **0.72 (0.60, 0.87)** |

* The unit of incidence density is per 100 person years

Model 1 was crude model;

Model 2 adjusted for age and sex;

Model 3 further adjusted for smoking and drinking status;

Model 4 further adjusted for HbA1c (<6.5, ≥ 6.5%), kidney disease, stroke, pulmonary disease, diabetic complications, prothrombin time (<10, 10-13, >13s), CYFRA21-1 (≤3.15, >3.15ng/mL), CA-125 (≤35, >35 U/mL), CEA (for non-smokers: <2.5, ≥2.5 μg/L; for smokers: < 5, ≥ 5 μg/L), chemotherapy, immunotherapy, targeted therapy, biguanides, surgical treatment, type of pathology, and metastasis (with, without).

Table S4. The association between GLP-1RA, DDP-4I, and SGLT-2I treatment duration and death (Sensitivity analysis).

| Therapy | Case/Total | Incidence density* | Model 1 | Model 2 | Model 3 | Model 4 |
| --- | --- | --- | --- | --- | --- | --- |
| **GLP-1RA** |  |  |  |  |  |  |
| Without | 687/4407 | 10.97 | 1.00 | 1.00 | 1.00 | 1.00 |
| Quartile 1 (<106 days) | 5/136 | 2.40 | **0.22 (0.09, 0.53)** | **0.35 (0.15, 0.85)** | **0.36 (0.15, 0.86)** | 0.46 (0.19, 1.12) |
| Quartile 2 (106-292 days) | 11/134 | 6.17 | 0.57 (0.32, 1.04) | 0.79 (0.44, 1.44) | 0.80 (0.44, 1.45) | 0.97 (0.53, 1.78) |
| Quartile 3 (293-559 days) | 6/138 | 3.12 | **0.29 (0.13, 0.65)** | **0.37 (0.16, 0.82)** | **0.37 (0.16, 0.82)** | **0.44 (0.19, 0.99)** |
| Quartile 4 (≥560 days) | 5/136 | 2.21 | **0.20 (0.08, 0.49)** | **0.28 (0.12, 0.68)** | **0.29 (0.12, 0.69)** | 0.49 (0.20, 1.19) |
| **DDP-4I** |  |  |  |  |  |  |
| Without | 687/4407 | 10.97 | 1.00 | 1.00 | 1.00 | 1.00 |
| Quartile 1 (<111 days) | 154/807 | 12.44 | 1.25 (0.95, 1.34) | 1.06 (0.89, 1.26) | 1.06 (0.89, 1.26) | 0.94 (0.79, 1.13) |
| Quartile 2 (111-302 days) | 152/809 | 13.15 | **1.20 (1.01, 1.43)** | 1.18 (0.993, 1.41) | 1.18 (0.99, 1.40) | 1.01 (0.84, 1.21) |
| Quartile 3 (303-622 days) | 122/810 | 9.66 | 0.87 (0.72, 1.06) | 0.89 (0.74, 1.08) | 0.88 (0.73, 1.07) | 0.86 (0.71, 1.05) |
| Quartile 4 (≥623 days) | 89/811 | 6.56 | **0.59 (0.47, 0.74)** | **0.61 (0.49, 0.76)** | **0.61 (0.49, 0.76)** | **0.69 (0.55, 0.86)** |
| **SGLT-2I** |  |  |  |  |  |  |
| Without | 687/4407 | 10.97 | 1.00 | 1.00 | 1.00 | 1.00 |
| Quartile 1 (<126 days) | 172/1159 | 10.67 | 0.98 (0.83, 1.16) | 0.96 (0.81, 1.13) | 0.95 (0.81, 1.13) | **0.91 (0.77, 1.08)** |
| Quartile 2 (126-321 days) | 172/1161 | 10.86 | 0.99 (0.84, 1.17) | 1.00 (0.84, 1.18) | 1.00 (0.85, 1.19) | 0.96 (0.81, 1.14) |
| Quartile 3 (322-607 days) | 150/1158 | 8.68 | **0.79 (0.66, 0.94)** | **0.82 (0.68, 0.97)** | **0.82 (0.69, 0.98)** | 0.85 (0.72, 1.02) |
| Quartile 4 (≥608 days) | 93/1162 | 4.80 | **0.43 (0.35, 0.54)** | **0.48 (0.39, 0.59)** | **0.48 (0.39, 0.60)** | **0.62 (0.50, 0.77)** |


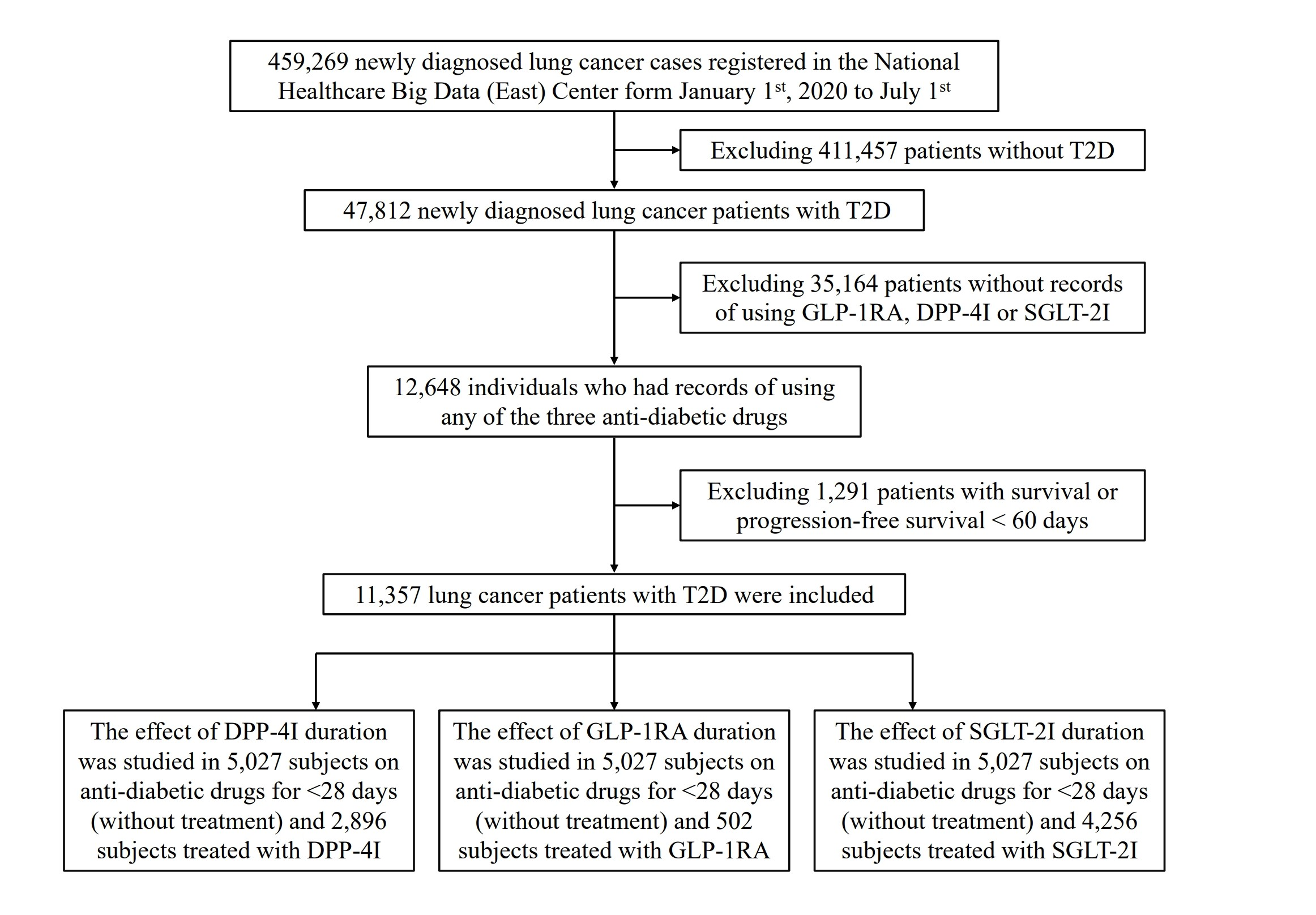


**Figure S1. Flow-chart (primary analysis)**

**
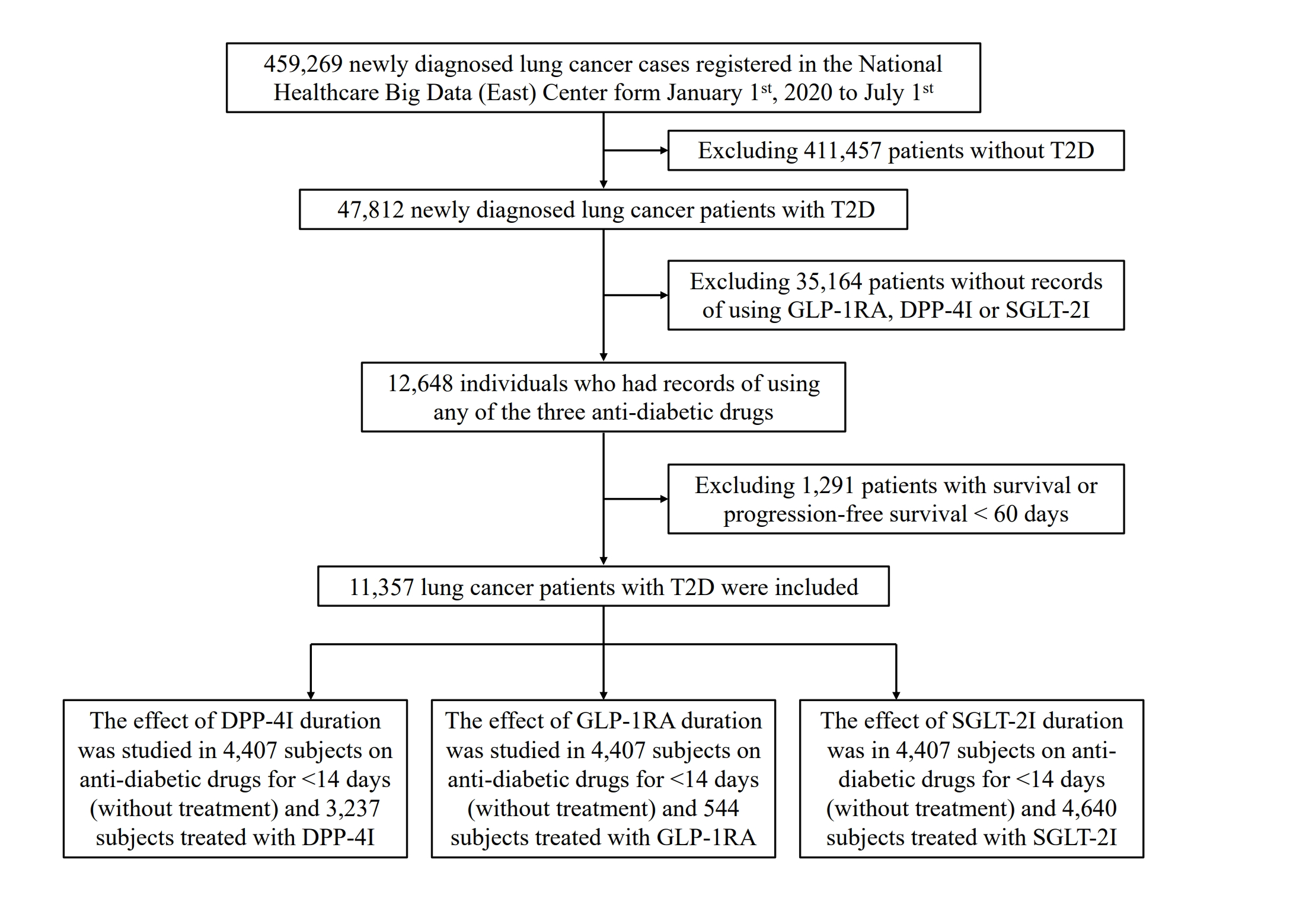
**

**Figure S2. Flow-chart (sensitivity analysis)**

**

**

**Figure S3. The dose-response relationship between DDP-4I (A&B), GLP-1RA (C&D), SGLT-2I (E&F) and the lung cancer progression and mortality.** A, C and E showed the dose-response relationship between treatment duration of 3 drugs and lung cancer progression, while B, D and F showed the dose-response relationship between treatment duration of 3 drugs and death (Sensitivity analysis).
